# Measuring Reasoning about Teaching for Graduate Admissions in Psychology and Related Disciplines

**DOI:** 10.3390/jintelligence5040034

**Published:** 2017-11-07

**Authors:** Robert J. Sternberg, Karin Sternberg, Rebel J. E. Todhunter

**Affiliations:** Department of Human Development, Cornell University, Ithaca, NY 14853, USA; karin.sternberg@gmail.com (K.S.); rjt93@cornell.edu (R.J.E.T.)

**Keywords:** reasoning, reasoning about teaching, reasoning about research, fluid abilities, successful intelligence

## Abstract

Teaching- and teaching-evaluation skills are critically important to professional success in psychology and related disciplines. We explored the possibility of measuring reasoning-about-teaching skills as a supplementary measure for admissions in psychology and related behavioral-sciences disciplines. We tested 103 students for their reasoning about teaching and their reasoning about research, as well as for their cognitive- (abstract reasoning) and educational skills. We found that women performed better than men on our reasoning-about-teaching measure, and that factorially, our reasoning-about-teaching measure clustered with our reasoning-about-research measures but not with our measures of abstract cognitive reasoning and educational skills.

## 1. Introduction

Graduate school in scientific psychology and related disciplines (e.g., human development, educational psychology, brain science, etc.) trains its students for two main tasks—scientific research and teaching. Traditionally, there has probably been more emphasis on research in graduate training, but for many if not most graduates, the large majority of their time will be spent fulfilling teaching responsibilities. Although many students have their sights set on tenure-track jobs in major research universities, the large majority of students who actually get academic jobs will land in teaching-intensive liberal-arts colleges, state-university campuses, or community colleges. Even among those who do make it to major research universities, many will be adjuncts whose paid responsibilities exclusively involve teaching assignments (see [1,2], for more details on these issues). Good teaching is critical not only for future scientists, but for the lives of the students that the scientists will impact through their instruction [3,4]. Scholarship in psychology and related disciplines should advance not only the research agenda of the university, but also the university’s teaching agenda [5].

Students who do not choose an academic career—or who are forced into a non-academic career through the dearth of academic jobs—may believe that they have no need to learn to teach. Chances are, they are wrong. In most of the jobs they go into—consulting, government research, nonprofits, amongst others—they will find themselves needing to make presentations to others [6]. Sometimes, the stakes for such presentations are even higher than for teaching a single class, such as a decision whether to award a consulting contract or the decision whether to begin the manufacture of a new product. So even for those students, oral-presentation skills can be critically important.

A previous set of investigations [7] explored the relationship between scientific reasoning skills, such as formulating scientific hypotheses, designing scientific experiments, evaluating scientific conclusions, and reviewing research articles, and to scores on conventional tests of cognitive abilities (e.g., letters sets and number series, which are tests of inductive-reasoning skills; and the SAT and ACT, which are tests of knowledge and analytical reasoning). It was found that the scientific-reasoning tests tended to correlate with each other, as did the conventional tests of cognitive abilities; however, the scientific-reasoning tests generally did not correlate significantly with the tests of cognitive abilities, and in some cases, correlated negatively. Those investigations suggested that tests of scientific reasoning might provide useful supplements to conventional tests of knowledge and analytical reasoning in graduate admissions, at least in psychology and related disciplines. Work on undergraduate admissions also suggested that conventional tests could benefit from supplementation with others kinds of measures, such as creative and practical thinking [8,9]).

The work on scientific reasoning only assessed research skills, not teaching or teaching-analysis skills. Yet as noted earlier, much of a typical scientific career is spent in teaching, preparing for teaching, or analyzing teaching. Analyzing teaching is particularly important, because it is through the analysis of others’ and one’s own teaching skills that one can become an excellent or at least good teacher [10]. Analyzing the quality of teaching is important not only for developing and improving one’s own teaching, but also for other tasks confronting faculty members. An analysis of teaching skills is typically involved, and should always be involved, in hiring new faculty members, promoting faculty members, tenuring faculty members, evaluating faculty members for raises, awarding teaching prizes, and other related activities. If one cannot analyze teaching (just as if one cannot analyze research), it is difficult to have a successful career as a faculty member.

Of course, being able to distinguish good and not so good teaching in oneself and others is not the only element in becoming a good teacher. One must also translate what one has learned into actual active teaching skills. Much of whether this translation will ever take place, however, will depend not only on a set of abilities, but also on the incentives of the institution and one’s motivation to become a good teacher. If one is teaching in a small liberal-arts college, the motivation to excel as a teacher will probably be very high; but if one is teaching in a large “Research 1” state university, the incentives for excellent teaching might be much lower. Thus, extrinsic and intrinsic motivational factors will probably be as important as analytical skills in determining who ultimately becomes a good teacher.

Because of the importance of being able to distinguish good from not so good teaching, it would seem reasonable to consider assessing such skills in the graduate-admissions process. From this point of view, if faculty members will spend a large proportion of their time teaching, do graduate programs really want to admit students who lack the skills necessary for successful teaching? Of course, students often start evaluating teaching as college students filling out course questionnaires, but research suggests that such evaluations are often very superficial and even wrong-headed [11,12,13,14,15,16].

There is not so much dispute over the kinds of behaviors that lead to good teaching in psychology and related disciplines [17,18]. These behaviors are regularly highlighted in journals such as *Teaching of Psychology* (http://journals.sagepub.com/home/top) through the American Psychological Association and *Psychology Teaching Review* (http://www.bps.org.uk/networks-and-communities/member-microsite/division-academics-researchers-teachers-psychology/psychology-teaching-review-ptr) through the British Psychological Society. These behaviors include topics like good organization, enthusiasm, moderate pacing, attention to the level of instruction, and so forth [10]. Of course, these also apply in fields other than psychology. Rather, the issue here is whether individuals can recognize both these elements of good teaching, and departures from them, when they experience them in instruction.

Our study was designed to assess people’s ability to evaluate, in an experimental situation, problematical behaviors in the teaching of psychology and related disciplines. The way in which we did this was to have professors with extensive teaching experience teach lessons in ways that were purposefully designed to embody particular flaws. The question then became whether the subjects viewing the teachers in the process of teaching would recognize their flaws in the teachers’ behavior. The flaws were not pointed out to the subjects. Rather, subjects had to reason about the teaching they were observing in real time.

There have been some attempts to look at teaching skills as a basis for admission to graduate school. For example, the University of Minnesota graduate application recommends applicants do a self-assessment of a number of skills, including ones pertaining to teaching (https://www.grad.umn.edu/current-students-academic-professional-development-building-your-plan/idpstep1). Questions they ask include:

- “Did you do any teaching in the past year (courses, seminars, laboratories)? Would you like additional opportunities to teach? How will you find these teaching opportunities?
- What sorts of feedback, formal or informal, have you received on your course content, syllabi, pedagogy, consideration of diverse learners and overall teaching abilities? In which areas do you need to improve? How will you improve your teaching and what resources are available?”

This assessment is a self-assessment, however, and it is not clear what its statistical properties are. Nor is it clear whether self-assessments are valid indicators of a person’s teaching skills or reasoning about teaching skills. We have been unable to locate specific formal instruments that have been used for graduate admissions that assess teaching skills for psychology. There are, of course, teacher-competency examinations, such as the Praxis (https://www.ets.org/praxis). There are three Praxis examinations. The Praxis Core Academic Skills for Educators (Core) tests measure reading, writing, and mathematic skills. The Praxis Subject Assessments measure subject-specific content knowledge, as well as general and subject-specific teaching skills, that individuals need for teaching. Moreover, the Praxis Content Knowledge for Teaching Assessments (CKT) measure subject-specific content knowledge, with an emphasis on content knowledge for K-12 teaching. The closest to what we are trying to accomplish is the Praxis Subject Assessment for teaching psychology (5391) in grades K-12. But these tests are of declarative knowledge, whereas our assessment is oriented toward procedural knowledge. Our assessment asks subjects to evaluate actual teaching as it is in progress, rather than to assess the teaching of psychology in the abstract.

We do not attempt here to fully review the extensive literature on assessments for graduate admissions. Such reviews can be found elsewhere (see, e.g., [7,19,20], for partial reviews). We present only a partial review.

The theoretical basis of our work is Sternberg’s [21,22,23,24,25,26,27,28,29,30,31] theory of successful intelligence. The theory holds that intelligence can be understood in terms of information-processing components that combine to produce intelligent behavior (see also [32,33]). When these components are implied insightfully, as in reasoning about scientific research and presumably about the teaching of science, they can solve problems that go beyond the usual kinds of routine problems that one confronts in everyday life [34,35]. The theory of successful intelligence is one of a class of theories that seek to understand intelligence in somewhat broader terms than conventional theories (see [36,37]; see also [38,39] for a theory arguing that conventional intelligence does not sufficiently take into account rational thinking). The theory of successful intelligence has been previously applied to undergraduate admissions [8], but only recently has been applied to graduate admissions [7] (see also [40]).

According to the theory [30], all analytical, creative, and practical abilities comprise the *same* components, namely, metacomponents, performance components, and knowledge-acquisition components. What differs in the three is how they are applied. The components of analytical abilities are executed in the application of the components to somewhat familiar but relatively abstract kinds of problems and situations. Creative abilities are executed in the application of these components to relatively novel tasks and situations. Practical abilities (as for evaluating teaching or research) are involved in the application of the components to those everyday concrete problems and situations with which one is confronted in one’s work and personal life. This means that analytical, creative, and practical abilities should show some correlation—they depend on the same information-processing components—but the extent of the correlation will vary depending upon the extent to which the contexts of measurement overlap. There is no one expected level of correlation but rather it depends on the particular tasks, situations, and their contextual overlap. It is difficult to specify in advance exactly what this overlap will be. For example, the practical abilities of a physicist may be more overlapping with the analytical abilities of a physicist than would be the case for an artist or a musician. In the theory of successful intelligence, there are no absolutely “pure tasks”, because so many tasks require a combination of analytical, creative, and practical skills.

Individuals can be strong in general abstract analytical skills but not necessarily strong in applying those skills to any one particular domain of practice. For example, someone might be adept at solving number or letter series, or at solving general mathematical problems, but not be adept when applying the same inductive reasoning skills to a domain of practice such as legal, medical, or scientific problem solving [29,40], or to reasoning about teaching, for that matter. The basic argument as it applies here is that the cognitive skills needed to succeed in teaching are in part different from the abstract analytical skills measured by tests such as the GRE (Graduate Record Examination). Abstract-reasoning skills certainly matter, but they are not the whole story. Thus, the goal here is not to supplant tests such as the GRE, but to examine whether future assessors would perhaps wish to look beyond such measures to measures that assess a person’s skill in evaluating the quality of teaching.

The basic question in our study is where the reasoning-about-teaching measure will place in terms of its factorial structure in relation to the other measures. There are various hypotheses about how it might load.

One hypothesis would be that reasoning about teaching is relatively different from reasoning about research, but it is inductive and draws on knowledge, so that the new reasoning-about-teaching measure should cluster with the tests of cognitive (abstract reasoning) and educational skills.

A second hypothesis would be that reasoning about teaching is basically similar to reasoning about research, in which case, factorially, the new reasoning-about-teaching measure would cluster with the reasoning-about-research measures.

A third hypothesis is that reasoning about teaching is similar neither to reasoning about research nor to traditional cognitive educational skills. In this case, the measure would cluster with neither of the other two groupings and instead would form its own factor. It might represent a set of skills entirely different from the skills measured by conventional tests or by measures of scientific reasoning.

The theory of successful intelligence would be consistent with either of the second or third hypotheses. Reasoning about teaching is a contextualized skill, so we would not expect it to be closely related to the kinds of reasoning on conventional tests of cognitive and educational skills. But whether this kind of reasoning is similar to reasoning about research was not clear to us in advance, and so we made no prior prediction regarding the second and third hypotheses. It might or might not be similar to reasoning about research.

There is one aspect of the experimental design that will be described below that might tend to yield to a separate reasoning-about-teaching factor. This is that whereas our reasoning-about-research measures are printed scenarios that can be read at a subject’s leisure and analyzed at whatever rate is comfortable for the subject, the reasoning-about-teaching measure is a video presentation in real time. Although subjects can take as long as they wish to provide responses, the videos themselves are rather quickly paced, and subjects have to watch them and then respond. If it turned out instead that, even with this methodological difference, the second hypothesis was nevertheless correct, it would suggest that reasoning about research and reasoning about teaching truly are rather closely bound.

## 2. Method

### 2.1. Participants

A total of 69 women and 34 men, 103 participants in all, were recruited for this study. All participants were current Cornell undergraduate and graduate students. They ranged in age from 18 to 30 years, and averaged 20 years of age; all were at least 18 years of age. The subjects were of diverse ethnicities, with seven African-American students, 43 Asian-American students, 33 European-American students, five Hispanic-American students, two mixed ethnicity students, eight other ethnicities, and five subjects who did not provide ethnicity data.

### 2.2. Materials

We used three kinds of assessments—a reasoning-about-teaching assessment, psychometric assessments measuring aspects of psychometrically defined general intelligence, and our own reasoning-about-scientific-research tasks--plus a demographic questionnaire. The psychometric tests were chosen to provide strong measures of discriminant validity for our measures. If our measures were assessing nothing more than general intellectual or educational skills, then there would be no need for them, as such tests already exist and are readily available.

#### 2.2.1. Reasoning about Teaching Video Assessment

Following a consultation with colleagues who teach lecture courses in psychology and related disciplines, the experimenters initially identified a potential list of flaws in teaching, namely: 1. Speaks too fast, 2. Speaks too slowly, 3. Lacks enthusiasm, 4. Lacks good organization, 5. Has not pretested failing equipment (LCD, computer), 6. Poor use of visuals, 7. Hard to understand what lecturer is saying, 8. Explanations are unclear, 9. Looks away from students, 10. Makes inappropriate jokes, 11. Intimidates or humiliate students, 12. Fails to answer questions asked, 13. Presents personal opinions as facts, 14. Monotone presentation, 15. Too abstract—does not give life-relevant concrete examples, 16. Appears not to know or understand the material, 17. Does not allow opportunity for questions, 18. Does not vary classroom style, 19. Lacks eye contact with students, 20. Teacher focuses attention on just a few students, and 21. Speaks too quietly or loudly. The flaws actually intentionally incorporated into each of the four videos are shown in Appendix A.

The subjects watched recordings of two professors, one male and one female (with 50 years of didactic teaching between them), from the Cornell College of Veterinary Medicine, each teaching two different short lessons. The female professor was 60 years old, and the male was 64 years old. Both were European-American. The videos were each 2.5–3 min long, with flaws purposely incorporated into the presentations. The four videos were on Piaget’s stage theory of development [41], egocentrism in young children [41], Ainsworth’s strange-situation problem [42], and Bronfenbrenner’s ecological systems theory [43]. All were presented by PowerPoint accompanied by the lecture of the male or female instructor, with each instructor presenting two of the four different lectures.

The instructors were given the list of teaching flaws (above). They were asked to impair their teaching, using this list, so that students could identify flaws in their teaching. The first two videos were presentations by the female professor. In her first lesson, for example, she purposely spoke too fast, gave unclear explanations, appeared to lack knowledge on the subject material, and was difficult to understand. In her second presentation, for example, she purposefully lacked enthusiasm, spoke to slowly and in monotone, and used visuals poorly, among other flaws. The second two videos were presentations given by the male professor. In his first lesson, for example, he failed to answer questions when asked, had trouble using PowerPoint equipment, and lacked organization. In his second lesson, for example, he presented personal opinions as facts, made inappropriate comments, and didn’t give any relatable examples. The subjects were asked to identify and write down all the flaws they observed in each lecture. Each student received a score based on how well he or she was able to evaluate the teaching flaws in the professors’ lectures. Subjects, of course, were not provided with the master list of flaws. Identification of flaws by the subjects occurred after each lecture.

The teaching task was new to this study (whereas all other measures in this study had been used by Sternberg & Sternberg [7]). Subjects received the following instructions:

“Students are frequently asked to judge the quality of teaching by professors. But often these judgments are holistic, reflecting general feelings about teaching rather than specific strengths or weaknesses. The following task provides you an opportunity to judge professorial teaching in an analytical way.

You will view four lectures, with two lectures given by each of two professors. The lectures were purposely designed to incorporate flaws in the professors’ teaching. We would like you to find those flaws. Examples of flaws would be “speaks too fast” or “doesn’t know material”.

On your answer sheet, you will see that space is allotted for each of the four lectures you will watch. Please view each lecture. As you watch and listen to the lecture, please list what you see as the flaws in the teaching. Each of the four lectures may have different flaws. Although each professor teaches two lectures, the flaws in the two lectures taught by each professor may be different. List as many flaws as you can. Please write legibly. Please also be clear in pointing out just what are the elements of each lecture that you believe are flawed. You will have some additional time after each lecture to finish your list of flaws.

Please note that the recordings of the lectures are imperfect. Do not deal with the quality of the video recordings. Deal only with the lectures and the lecturers themselves.

Thank you.” 

Blank spaces were provided for subjects to fill in the errors after they were presented with each lecture. Scoring was based on the number of errors correctly identified as discussed below.

The reliability in the current study of this measure correlating scores for ratings of Professor 1 with ratings of Professor 2, corrected by the Spearman-Brown formula (because each represented only half the assessment), was 0.73. (This is a conservative estimate because the two professors were not truly “equivalent” halves of the assessment.)

#### 2.2.2. Psychometric Assessments

Subjects were presented with a questionnaire that assessed their analytical abilities by means of two subtests: (a) a letter-sets test, in which they had to choose a set of letters that did not fit in with other sets of letters presented; and (b) a number-series test, in which they had to find the correct number to continue the number series presented to them. We created the tests ourselves and validated them in Sternberg and Sternberg [7]. The two tests are largely measures of fluid intelligence (Carroll, [44]).

**Letter sets.** The letter-sets test consisted of 15 problems. The participants were asked to identify patterns among sets of letters and eliminate the group of letters that did not fit the pattern. They had seven minutes to complete as many of the problems as they could. For example, if the first problem gave the letter groups of CDEF, HIJK, QRST, IJKM, and OPQR, IJKM would have been eliminated because it is the only group of letters that is not in alphabetical order. The subjects would have answered this correctly if they drew a cross over IJKM.

**Number series.** The second number-series test presented items that contained a series of numbers. Subjects were asked to find the number that should have appeared next in the series. This test contained 18 problems. The subjects were given seven minutes to complete as many of the problems as they could. An example of one problem would be if the student was presented with the number series, 2, 4, 6, 8, …… In this example, each number is derived by adding 2 to the previous one. Therefore, the correct solution is 10.

#### 2.2.3. Scientific-Reasoning Assessments

The scientific-reasoning tasks were chosen because they represent thinking processes—generating hypotheses, generating experiments, drawing conclusions, reviewing—that are necessary (although not sufficient) for scientific research (Sternberg & Sternberg [7]).

**Generating hypotheses.** Subjects were presented with short descriptions of situations and had to create alternative hypotheses to explain the behavior described in the vignettes (see Appendix A). One vignette said, for example:

Marie is interested in child development. One day, she notices that whenever Laura’s nanny comes in to pick up Laura from nursery school, Laura starts to cry. Marie reflects upon how sad it is that Laura has a poor relationship with her nanny.

What are some alternative hypotheses regarding why Laura starts to cry when she is picked up from nursery school by the nanny?

The interrater reliability of this assessment has previously been found to be 0.96 (Sternberg & Sternberg [7]).

**Generating experiments.** A second set of vignettes described a situation with hypotheses, and students were asked to design an experiment to test these hypotheses. Here is an example:

Ella, a senior in college, observes that her roommate tends to perform better on an exam if she has had a cup of coffee beforehand. Ella hypothesizes that drinking coffee before taking an exam will significantly increase one’s exam performance. However, Ella does not know how to test this hypothesis.

Please suggest an experimental design to test this hypothesis and describe the experiment in some detail. Assume you have the resources you need to be able to do the experiment (e.g., access to students and their academic records, sufficient funds to pay subjects, etc.).

The interrater reliability of this assessment has previously been found to be 0.80 [7].

**Drawing Conclusions.** A third set of vignettes presented students with the results of studies and asked whether the conclusions drawn were valid. Items looked like the following:

Bill was interested in how well a new program for improving mathematical performance worked. He gave 200 students a pretest on their mathematical knowledge and skills. He then administered the new program to them. After administering the program, he gave the same 200 students a posttest that was equal in difficulty and in all relevant ways comparable to the pretest. He found that students improved significantly in performance from pretest to posttest. He concluded that the program for improving mathematical performance was effective.

Is this conclusion correct? Why or why not?

The interrater reliability of this assessment has previously been found to be 0.71 (Sternberg & Sternberg, [7]).

**Reviewing***.* In this item type, comprising a single item, students had to play the role of a reviewer of a journal article. They read a two-page description of an experiment and were then asked to indicate the article’s flaws and problems. Answers were given in the style of an essay, much as would be a review of a journal article written for a journal editor. In one of the items, participants read a short report on the validation of an extraversion scale. After reading the report, they were asked: “Is the article you just read adequate or not? If not, what are the article’s flaws and problems?” 

The interrater reliability of this assessment has previously been found to be 0.97 (Sternberg & Sternberg [7]).

Overall, the interrater reliability of the measures previously used has been found to be 0.91 for generating hypotheses, generating experiments, and drawing conclusions, together, and the average reliability of 0.93 was identified for the full composite of assessments with the reviewer included (Sternberg & Sternberg [7]).

#### 2.2.4. Demographic Questionnaire

A demographic questionnaire assessed variables like gender, age, major, race, and SAT/ACT scores, as well as GPAs. Two other questions were asked but numbers were too small to analyze the data, in particular, college major and subjective comments on one’s past research experience.

### 2.3. Design

The independent variables for the analysis of variance were demographic characteristics, in particular, male versus female subjects. Our main concern was with gender, as we did not have enough subjects in the other breakdowns to do separate statistical analyses.

The dependent variables were scores on the various assessments we gave. For the factor analyses, the independent variables were the hypothetical factors and the dependent variables were the various assessments we gave. That is, in theory at least, latent factor scores predict observable scores on tests.

### 2.4. Procedure

Subjects participated in the study in groups of up to 12 subjects. First, they read and signed an informed-consent form. Then, they were handed out questionnaires. The assessments were arranged in the questionnaire in the following order: Letter Sets test, Number Series test, teaching analysis, generating hypotheses, generating experiments, drawing conclusions, reviewing, demographic questionnaire. The first two tests were timed, so the students were guided together through those two assessments by an experimenter with a stopwatch. None of the scientific-reasoning or reasoning-about-teaching exercises had a time limit.

When the subjects had completed all the materials, they returned them to the experimenter, who presented them with a debriefing form. Students read the form and indicated with a signature whether or not their data could be used in our data analysis. Then they received compensation for their participation in the study in the form of either course credit or payment ($20). All subjects were compensated in one way or the other.

Finally, we gave participants a written debriefing. The entire study took about 90 min and was conducted in person.

## 3. Results

All assessments were scored-based either on objective answer keys (i.e., Letter Sets and Number Series) or rubrics (i.e., all other assessments; see also [7]). Because our task inter-rater reliabilities were very high in our previous work, ranging from 0.71 to 0.97 with a median of 0.88 [7], we only used one trained rater (a psychology PhD) in the present study.

In all studies, scoring was for the rater judgment of the number of correct answers. No exact wording was required—scoring was based on concepts. If an answer was repeated (including with slightly different wording), it was not scored as correct the second (or any subsequent) time. For the teaching measure, if a subject pointed out a flaw not intentionally introduced into the lecture, a judgment was made as to whether the answer was justifiable. The wording did not matter so long as the subject got the idea of the flaw and was specific. Thus, “spoke too fast” or “read from a piece of paper,” or “did not answer questions” would count as identified flaws, but “didn’t know how to teach,” “struggled to convey the material,” or “had some kind of accent” would not get credit. Details on scoring from measures in Sternberg and Sternberg [7] are presented in that article.

### 3.1. Basic Statistics

Basic statistics for the study are shown in Table 1. We had no specific hypotheses regarding gender. None of the differences were significant (see Table 2).

### 3.2. Correlations

Table 3 shows, for the full sample, the intercorrelations between the variables of main interest in our study. Of greatest interest is the fact that our new teaching-flaws assessment did not correlate significantly with the standardized tests of cognitive skills (and indeed tended to show negative correlational trends, as also found in Study 1 of Sternberg and Sternberg [7]), but did correlate positively with generating hypotheses, drawing conclusions, and reviewing. Contrary to our expectation, it did not correlate significantly with generating experiments.

Table 4 shows the intercorrelations of the students’ response to the four teaching scenarios. They ranged from 0.25 to 0.48 with a median of 0.41. All were statistically significant, as one would expect.

The correlation of responses across the two lecturers (male and female) was 0.57 (*p* < 0.01). These results, combined, suggest that students who were adept at recognizing flaws in one lecture were adept at recognizing flaws in other lectures, and that students who were not adept at recognizing flaws for one lecturer were not adept at recognizing flaws for the other lecturer. In other words, ratings of the various items and lecturers seem to have been measuring pretty much the same basic construct.

### 3.3. Principal Components and Factor Analyses

Table 5 and Table 6 show, for the full sample, principal-components and maximum-likelihood factor analyses of our measures of interest, using a minimum Eigenvalue of 1 criterion as the basis for choosing the number of factors. As in Sternberg and Sternberg [7], the two factor analyses gave similar results but the principal-components analysis with varimax rotation is somewhat more easily interpretable. Factor I represents the standardized tests of cognitive abilities and educational skills, which are all likely to be largely *g* loaded [45]. Factor II represents Generating Hypotheses, Drawing Conclusions, and Teaching (and to some extent Reviewing). Factor III represents Generating Experiments and Reviewing. Thus, the standardized tests group on Factor I, and our new assessments for reasoning about research and teaching groups on Factors II and III. Thus, the results largely support the second hypothesis—that the reasoning-about-research and reasoning-about-teaching assessments measure similar skills. We are not clear on why, in this study, our new measures split into two factors. However, hypothesis generation, uniquely among the new assessments, involved highly divergent thinking and students were given leeway in responses so long as any given hypothesis was justifiable.

## 4. Discussion

Our goal in this study was to explore the feasibility of adding a measure of evaluation of scientific teaching into the graduate-admission process. In particular, we were interested in whether a new measure of reasoning about teaching—evaluation of flaws in teaching—would prove itself to cluster factorially with traditional tests of cognitive and educational skills (the first hypothesis), would prove to cluster with our reasoning-about-research measures (the second hypothesis), or would form its own factor (the third hypothesis). Our data best fit the second hypothesis. Factorially, our measure of reasoning about teaching flaws clustered with hypothesis generation, drawing conclusions, and reviewing research. In this study, the reasoning-about-research measures clustered into two different factors (in contrast to Sternberg & Sternberg, where they formed a unified factor). Our measure of reasoning about teaching did not correlate with any of the research-evaluation measures at a level anywhere close to the reliabilities of the respective measures, suggesting that it possessed a unique variance of its own that was not shared with the research-evaluation measures. Thus, it is a useful supplement to those measures, rather than being redundant with them.

The study does not look at the modifiability of reasoning about flaws in teaching or of reasoning about scientific research. Almost certainly these skills can be taught [46,47], and books on experimental methods (e.g., [48,49]) try to teach these skills, as do books teaching critical thinking [50,51].

Our study, of course, had various limitations.

First, our subjects were all undergraduates at Cornell. Although Cornell is more diverse than any of the other Ivy League Schools (containing separate colleges for disciplines as diverse as arts and sciences of business, hotel administration, agriculture, human ecology, engineering, and industrial and labor relations), there certainly would be some restriction of range in abilities beyond that of students applying to attend graduate school in psychology and related disciplines.

Second, our instruction was limited to four topics in developmental psychology. Although the topics themselves were somewhat diverse, they did not include many of the areas of psychology, for example, brain science or humanistic psychology.

Third, our sample was only slightly more than 100 subjects, and thus not particularly large. Future research would need a larger sample of subjects.

Fourth, although all of our measures have been used before (Sternberg & Sternberg, [7]), except for the reasoning-about-teaching measure, their use has not been extensive. We do not have extensive prior data on these measures.

Fifth, we were measuring reasoning about teaching, not actual teaching. In a subsequent study currently being run, we actually have students give a brief lecture. We believe this follow-up is important because there is no guarantee that someone who is good at spotting flaws in lectures is also good in lecturing (and vice versa). However, most students have had very limited experience in lecturing, and so it is not clear how predictive we can expect their own teaching to be when they are so inexperienced in teaching. Such a task may prove to be beyond the students’ zone of proximal development (Vygotsky [52]). Moreover, such a task probably heavily involves extrinsic and intrinsic motivational factors that may have little to do with subjects’ actually teaching, learning, or reasoning abilities [53,54].

Finally, and perhaps most importantly, our subjects were not ones specifically targeting graduate school in psychology and related disciplines, and we cannot say how they would have performed in graduate school. Even if we targeted applicants to graduate schools in psychology and related disciplines, it would have been difficult to follow up, because the students then would have gone to very diverse schools with widely varying systems of evaluation, and it is not clear how we truly could have kept track of them.

What our study did suggest, however, is that it appears to be possible to measure students’ reasoning about teaching flaws; an important skill students need to succeed not only in academia, but in the world of science, more generally. Our new measure is certainly not a finalized instrument, but it suggests that a more refined instrument could be developed based on students watching lectures (or other classroom situations) and commenting on the flaws (and perhaps in future assessment, the strengths) in specific situations. Teaching is critically important to success in many jobs in psychological and related sciences, and a measure that assesses skills relevant to success in teaching certainly deserves consideration as a supplementary measure for admission in graduate education in the psychological sciences and related disciplines. Most importantly, the current research suggests the importance of scholarship that advances the teaching as well as the research agenda of the university (Boyer [5]). If the goal of a university is to develop the active concerned citizens and ethical leaders of the next generation (Sternberg [9]), then this can only be done if the university seeks to admit and then educate the best teachers that our society can possibly find. Such teachers will excel not only in the analytical skills measured by conventional standardized tests, but also in the practical skills of the intellect [55,56,57,58,59] that are so important in teaching and other professions as well as in the creative skills involved in teaching and being a serious scientist [60].

## Figures and Tables

**Table 1:** Basic statistics.

Variable	Mean	Standard Deviation	N
Age	20.04	2.32	103
Year in College	2.41	1.18	103
Cumulative College GPA	3.51	0.38	78
SAT Reading	700.00	78.32	73
SAT Math	722.57	93.11	74
ACT Reading	32.89	2.57	44
ACT Math	33.09	2.73	45
Number of Lab Courses	1.66	2.41	97
Research Methods	1.66	0.48	100
Letter Sets Score	10.19	2.57	103
Number Series Score	10.78	3.51	103
Hypotheses Score	7.43	3.51	103
Experiments Score	6.07	1.32	103
Conclusions Score	6.09	1.31	103
Reviewer Score	7.28	3.54	103
TEACHING SCORE	17.44	3.87	101

**Table 2:** Multivariate analysis of variance.

Descriptive Statistics											
		**Gender**				**Mean**		**Std. Deviation**		**N**	
Hyp		Male				7.39		4.02		33	
		Female				7.49		3.26		68	
		Total				7.46		3.51		101	
Exp		Male				6.06		1.27		33	
		Female				6.12		1.33		68	
		Total				6.10		1.31		101	
Concl		Male				6.09		1.31		33	
		Female				6.13		1.31		68	
		Total				6.12		1.31		101	
Reviewer item correct responses		Male				7.27		3.12		33	
		Female				7.43		3.71		68	
		Total				7.38		3.51		101	
Teach		Male				17.00		3.58		33	
		Female				17.66		4.02		68	
		Total				17.45		3.87		101	
**Multivariate Tests ^a^**											
**Effect**			**Value**		**F**		**Hypothesis df**		**Error df**		**Sig.**
Wilks’ Lambda			0.99		0.14 ^b^		5.00		95.00		0.983
**Tests of Between-Subjects Effects**											
**Source**	**Dependent Variable**			**Type III Sum of Squares**		**df**	**Mean Square**		**F**		**Sig.**
Corrected Model	Hyp			0.18 ^c^		1	0.18		0.015		0.903
	Exp			0.07 ^d^		1	0.07		0.042		0.838
	Concl			0.04 ^e^		1	0.04		0.022		0.882
	Reviewer item correct responses			0.52 ^f^		1	0.52		0.042		0.838
	Teach			9.73 ^g^		1	9.73		0.646		0.423
Gender	Hyp			0.18		1	0.18		0.02		0.903
	Exp			0.07		1	0.07		0.04		0.838
	Concl			0.04		1	0.04		0.02		0.882
	Reviewer item correct responses, rater 1			0.52		1	0.52		0.04		0.838
	Teach			9.73		1	9.73		0.65		0.423
Error	Hyp			1230.86		99	12.43				
	Exp			170.94		99	1.73				
	Concl			170.54		99	1.72				
	Reviewer item correct responses			1231.18		99	12.44				
	Teach			1491.22		99	15.06				
Total	Hyp			6845.00		101					
	Exp			3928.00		101					
	Concl			3952.00		101					
	Reviewer item correct responses			6727.00		101					
	Teach			32240.00		101					
Corrected Total	Hyp			1231.05		100					
	Exp			171.01		100					
	Concl			170.57		100					
	Reviewer item correct responses			1231.70		100					
	Teach			1500.95		100					

^a^ Design: Intercept + Gender; ^b^ Exact statistic; ^c^ R Squared = 0.000 (Adjusted R Squared = −0.010); ^d^ R Squared = 0.000 (Adjusted R Squared = −0.010); ^e^ R Squared = 0.000 (Adjusted R Squared = −0.010); ^f^ R Squared = 0.000 (Adjusted R Squared = −0.010); ^g^ R Squared = 0.006 (Adjusted R Squared = −0.004).

**Table 3:** Correlations of Selected Variables.

Variable	GPA	SAT-R	SAT-M	ACT-R	ACT-M	Res. Exp.	Number of Labs	Res. Meth.	Let. Sets	Numb. Series	Hyp. Gen.	Exp. Gen.	Conclusions	Reviewer	Teaching
GPA	1.00		0.17	0.05	0.00	0.27 *	−0.24 *	−0.14	0.16	−0.02	−0.15	0.05	0.00	0.12	−0.10
SAT-R		1.00	0.39 **	0.66 **	−0.06	0.00	−0.18	−0.10	0.12	0.25 *	0.02	0.11	0.03	0.42 **	−0.04
SAT-M			1.00	−0.17	0.85 **	0.17	−0.23	−0.15	0.22	0.40 **	0.01	−0.01	0.00	0.17	
ACT-R				1.00	0.09	0.05	−0.02	0.12	0.22	0.06	0.15	0.40 **	0.32 *	0.05	−0.10
ACT-M					1.00	0.09	0.09	0.02	0.17	0.49 **	−0.06	−0.03	0.03	0.00	−0.19
Res. Exp.						1.00	0.50 **	−0.22	0.09	0.12	−0.23	0.05	0.09	−0.04	−0.13
Number of Labs							1.00	−0.08	−0.09	−0.06	−0.13	0.06	0.00	−0.24 *	0.00
Res. Meth.								1.00	0.10	0.07	0.07	−0.05	−0.05	−0.04	−0.12
Letter Sets									1.00	0.36 **	0.05	0.03	0.12	0.12	0.12
Numb. Series										1.00	0.07	−0.12	0.05	−0.05	−0.17
Hyp. Gen.											1.00	0.15	0.46 **	0.26 **	0.42 **
Exp. Gen.												1.00	0.42 **	0.24 *	0.13
Conclusions													1.00	0.32 **	0.30 **
Reviewer														1.00	0.30 **
Teaching															1.00

* *p* < 0.05; ** *p* < 0.01.

**Table 4:** Intercorrelations of the Students’ Responses to the Four Lectures.

Correlations					
		**Lecture 1**	**Lecture 2**	**Lecture 3**	**Lecture 4**
Teaching Item 1	Pearson Correlation	1	0.41 **	0.46 **	0.25 *
	Sig. (2-tailed)		0.00	0.00	0.01
	N	101	101	101	101
Teaching Item 2	Pearson Correlation	0.41 **	1	0.48 **	0.41 **
	Sig. (2-tailed)	0.00		0.00	0.00
	N	101	101	101	101
Teaching Item 3	Pearson Correlation	0.46 *	0.48 **	1	0.35 **
	Sig. (2-tailed)	0.000	0.000		0.000
	N	101	101	101	101
Teaching Item 4	Pearson Correlation	0.25 *	0.41 **	0.35 **	1
	Sig. (2-tailed)	0.01	0.00	0.00	
	N	101	101	101	101

** Correlation is significant at the 0.01 level (2-tailed). * Correlation is significant at the 0.05 level (2-tailed).

**Table 5:** Principal-Components Analysis with Varimax-Rotated Factors.

Variable/Factor	I	II	III
Hyp. Gen.	0.04	0.91	−0.09
Exp. Gen.	0.11	0.06	0.81
Conclusions	0.13	0.68	0.43
Reviewer	−0.12	0.36	0.55
TEACHING	−0.17	0.67	0.17
SAT-M	0.79	−0.06	−0.18
SAT-R	0.66	−0.03	0.21
Letter Sets	0.50	−0.07	0.23
Number Series	0.70	0.11	−0.38

Note: Total variance explained = 57.74%. Eigenvalues for the three rotated factors were 1.88, 1.88, 1.45, respectively.

**Table 6:** Maximum-Likelihood Principal-Factor Analysis with Varimax-Rotated Factors.

Variable/Factor	I	II	III
Hyp. Gen.	0.99	0.07	0.08
Exp. Gen.	0.02	0.06	0.63
Conclusions	0.47	0.11	0.59
Reviewer	0.23	−0.16	0.39
TEACHING	0.42	−0.17	0.25
SAT-M	−0.04	0.76	−0.11
SAT-R	−0.02	0.48	0.09
Letter Sets	−0.14	0.31	0.15
Number Series	0.07	0.56	−0.19

Note: Total variance explained = 42.28%. Eigenvalues for the three rotated factors were 1.47, 1.29, 1.04, respectively.

## Appendix A

### Teaching Flaws Incorporated into Videos

All Videos

1. Failed to vary the classroom style.
2. Failed to walk around or otherwise make an effort to actively engage with students.
3. Never asked if there were any questions and the male professor failed to answer questions when they were asked.
4. Read off a sheet of paper and did not attempt to hide it.

Video 1: Female Professor

1. Spoke too fast
2. It was hard to understand what lecturer was saying
3. Appeared not to know or understand the material
4. Lacked eye contact with students
5. Generally ignored students

Video 2: Female Professor

1. Spoke too slowly
2. Lacked enthusiasm
3. Used monotone presentation style
4. Checked her phone during the presentation
5. Seemed very uninterested in the material)

Video 3: Male Professor

1. Lacked organization
2. Gave a confusing presentation
3. Had not pretested failing equipment
4. Failed to make adequate contact (visually and otherwise) with students
5. Failed to answer questions asked

Video 4: Male Professor

1. Made inappropriate comments
2. Intimidated or humiliated students when they asked a question
3. Gave confusing examples
4. Gave examples that were not relevant
5. Presented personal opinions as facts
